# Nutrition Labelling Practices and the Healthiness of Packaged Food and Beverage Products Available in Kenya

**DOI:** 10.3390/nu18040566

**Published:** 2026-02-09

**Authors:** Elizabeth K. Dunford, Laura Kiige, Moeno Sakai, Agnes Erzse, Ismael Ngnie-Teta, Leah Richardson

**Affiliations:** 1 Department of Nutrition, Gillings Global School of Public Health, The University of North Carolina at Chapel Hill, Chapel Hill, NC 27514, USA; 2 Food Policy Division, The George Institute for Global Health, University of New South Wales, Sydney, NSW 2031, Australia; 3 UNICEF Kenya Country Office, Nairobi 00100, Kenya; lkiige@unicef.org (L.K.); msakai@unicef.org (M.S.); ingnieteta@unicef.org (I.N.-T.); lrichardson@unicef.org (L.R.); 4 UNICEF Eastern and Southern Africa Regional Office, Nairobi 00100, Kenya; aerzse@unicef.org

**Keywords:** nutrition labelling, nutrient profiling, processed foods

## Abstract

**Background/Objectives**: Kenya’s diet-related non-communicable disease burden is rising alongside the consumption of ultra-processed foods. Kenya finalized a national nutrient profile model (KNPM) in 2025, drawing on the WHO African regional model (WHO NPM). The objective of this study was to examine labelling practices and the healthiness of packaged products available in Kenya, including domestically produced and imported items, to identify policy priorities to strengthen nutrient profiling, surveillance, and alignment with international standards. **Methods**: Packaged food and beverage data were obtained from Innova Market Insights. The proportion of products meeting minimum Codex nutrition labelling requirements was determined. The proportion of products that met the nutrient criteria set out under the KNPM (draft and final versions) and WHO NPM was examined overall and by category. Agreement between nutrient profile models was determined using Fleiss’ kappa. **Results**: Of 5587 products, 21% displayed minimum Codex nutrient requirements. Labelling was more complete among imported compared to domestic products (40% vs. 14%). Sales-weighted eligibility was low: 15% (WHO NPM and draft KNPM) and 17% (final KNPM). Agreement across models was 82% (*k* = 0.44) and was highest between the WHO NPM and the final KNPM (95%; *k* = 0.66). Beverage patterns reflected stricter thresholds in the WHO NPM and the final KNPM. **Conclusions**: Kenya’s packaged food supply is inadequately labelled, with a large proportion not meeting the nutritional requirements set out in the final KNPM or WHO NPM. Mandatory, Codex-aligned nutrition labelling is necessary to ensure full operationalization of the KNPM, with regular review to reflect evolving food environments.

## 1. Introduction

The rate of overweight and obesity has increased among children and adolescents in low- and middle-income countries over the past decade and this increase is largely driven by unhealthy diets [1]. Poor diet, which to a large extent is driven by unhealthy food environments, now generates more non-communicable disease than physical inactivity, harmful use of alcohol and smoking combined [2]. The rise in diet-related non-communicable disease in Africa is strongly linked to an increasing consumption of store bought processed packaged foods that are often high in risk-associated nutrients such as sodium, saturated fat, trans fats and sugars [3]. In Kenya, the rise in supermarket shopping has led to increased consumption of processed and packaged foods [4]. Kenya has one of the most prospering supermarket sectors in sub-Saharan Africa [5]. Research suggests that between 2002 and 2018, grocery sales increased an average 12% per year [6], and increased supermarket purchases have been associated with higher body mass index in Kenya, as well as other countries in Africa [5].

The World Health Organization (WHO) recommends that food manufacturers reduce the levels of harmful nutrients in packaged food and beverage products to promote healthy diets and reduce the burden of diet-related non-communicable disease [7]. Nutrient profiling is the science of classifying or ranking foods according to their nutritional composition for the purpose of preventing disease and promoting health. Nutrient profile models (NPM) have been developed by academics, government departments, health-related charities and the food industry for a variety of applications including: to underpin nutrition labelling; to regulate advertising of products to children; and to regulate health and nutrition claims [8]. For the African region, there are a number of NPMs in use. In 2019, the WHO Regional African Office released their NPM (hereafter WHO NPM) aiming to support countries across Africa in their efforts to control obesogenic food environments and promote healthy diets, the primary focus being to protect children from the marketing of unhealthy foods and non-alcoholic beverages [9]. In 2021, the government of Kenya released a draft NPM, (hereafter draft KNPM), aiming to underpin national front-of-package nutrition labelling standards. An iterative process of refinements then took place over the ensuing few years, resulting in a final KNPM released by the government in July 2025 [10].

Whilst there has been research undertaken to assess the extent of implementation of food environment policies in Kenya and prioritize actions for creating healthier food environments [11], to date little research has assessed the overall nutritional quality and composition of the packaged food and beverage supply. As such, this is a critical research gap for the development, implementation and evaluation of healthy food environment policies, particularly setting and monitoring nutrition standards such as labelling and food composition. The objective of this study was to examine labelling practices and the healthiness of packaged products available in Kenya, including domestically produced and imported items, using regionally applicable NPMs. Results will help form a baseline from which progress can be monitored.

## 2. Materials and Methods

### 2.1. Data Sources

Packaged food and beverage data for this project derived from Innova Market Insights [12]. All packaged food and beverage products introduced to the Kenyan market between 2012 and 2021 were included in this report. Data from Innova Market Insights were categorized into one of 27 Euromonitor International categories to facilitate sales-weighting of results. All results were presented overall and by Euromonitor category. Products were flagged as outliers if they were above or below five standard deviations from the overall mean for total sugars, sodium, saturated fat or energy content. However, visual examination of each outlier flagged was undertaken to determine whether it should be removed from analysis. Any product missing critical information required for evaluation under each nutrient profile model was also excluded (see Table S1).

### 2.2. Assessment of Nutrition Labelling Practices

Overall and for each Euromonitor International category, the proportion of packaged food and beverage products displaying the minimum labelling requirements set out by Codex (energy, protein, carbohydrate, total fat, saturated fat, total sugar and sodium) plus nutrients of public health concern (trans fat) was examined. Results were also split into imported and domestically produced products and examined separately. Domestic products were defined as products with labels that specified they were manufactured in Kenya. Imported products were defined as products with labels that specified they were not manufactured in Kenya.

### 2.3. Assessment Against Existing Nutrient Profile Models

#### 2.3.1. WHO NPM

The NPM for the African region builds on the models developed in the other WHO regions [9]. It adopts the threshold approach, adapting food categories from other regions and incorporating foods that are commonly consumed in Africa. The model is presented in tabular format and consists of 18 categories (with 10 subcategories) of processed foods and components that are subject to restriction, namely, total and saturated fats, total and added sugars, sodium and energy. For this study, all products were assigned to a WHO NPM category.

#### 2.3.2. Draft and Final Kenyan Nutrient Profile Models

The draft KNPM was developed in 2021 to underpin front-of-pack nutrition labelling requirements in Kenya. The draft model included criteria concerning the levels of total fat, saturated fat, total sugars and sodium and proposed 21 foods and beverage categories to be subject to restriction based on varying nutritional criteria. The final KNPM released in July 2025 consisted of 25 categories [10]. For this study, all products were assigned to both a draft and final KNPM category.

### 2.4. Data Analysis

Data were analyzed using Stata V18 (Stata Corp., College Station, TX, USA). The proportion of products labelling the minimum nutrients as required under Codex, both overall and by import status (imported or domestically produced) was assessed. The proportion of products eligible under the WHO NPM, draft KNPM and final KNPM was examined both overall and by Euromonitor category. The proportion of products meeting eligibility criteria under each NPM was multiplied by category-level sales values (KES billion) from Euromonitor to estimate the proportion of revenue deriving from products that met the NPM criteria. Sales-weighting was based on category-level value sales (KES billion) from Euromonitor (i.e., monetary value), not unit volumes. Agreement among the three NPMs was examined using Fleiss’ kappa statistic. Kappa agreement was classified as follows: 0.01–0.20, slight agreement; 0.21–0.40, fair agreement; 0.41–0.60, moderate agreement; 0.61–0.80, substantial agreement; 0.81–0.99, near perfect agreement. Agreement assessment was restricted to include only those products with sufficient nutrient information to be examined under all three NPMs (see Table S1 for further details). Sales-weighted proportions were calculated overall, based on each category’s sales relative to the total combined sales of all packaged food and beverage categories. This approach was taken to apply a weighting that is most relevant for health impact (assuming sales are correlated with consumption). Foods and beverages were examined both together and separately.

## 3. Results

### 3.1. Assessing Nutrition Labelling Practices

Table 1 shows the proportion of packaged food and beverage products in Kenya displaying both the minimum labelling requirements set out by Codex as well as nutrients of public health concern. Overall, 21% of products displayed the minimum requirements set out by Codex, and only 4% labelled both the minimum requirements as well as key nutrients of public health concern. *Carbohydrate*, *Total fat* and *Protein* were the nutrients with the highest labelling compliance (60%). *Trans fat* was the least likely nutrient to be labelled (12%) followed by *Saturated fat* (37%) and *Total sugars* (38%). The *Soup* category had the highest labelling compliance with minimum Codex requirements (86%), followed by *Carbonates* (53%) and *Breakfast Cereals* (51%). *Breakfast Cereals*, *Edible Oils* and *Soup* were the only categories to have more than 10% of products displaying all nutrients examined.

Tables S2 and S3 examine the differences between imported and domestically produced/Kenyan products with regard to Codex labelling requirements. Overall, a much lower number of products were imported compared to domestically produced (*n* = 1406 versus *n* = 4181). Imported products were more likely than Kenyan products to display both the minimum Codex labelling requirements (40% versus 14%) as well as additional nutrients of public health concern (7% versus 3%). Category-level results for both imported and Kenyan products were similar to the overall results.

### 3.2. Assessing the Proportion of Products Eligible Under Each Nutrient Profile Model

**Figure 1:**
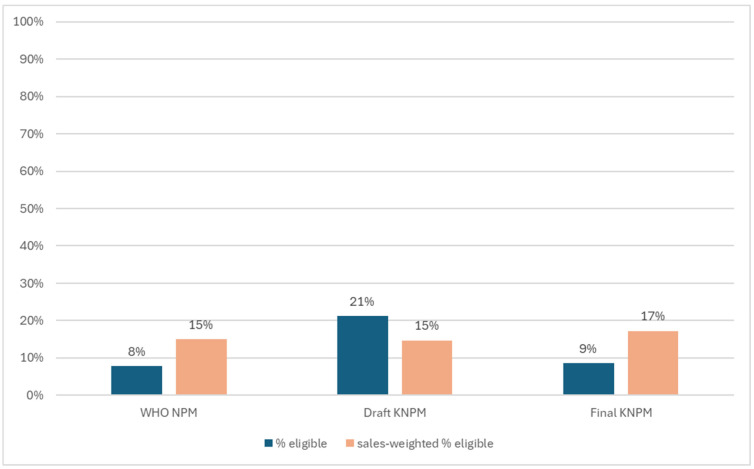
Proportion of products eligible under the WHO Nutrient Profile Model (WHO NPM), draft Kenyan Nutrient Profile Model (Draft KNPM), and final Kenyan Nutrient Profile Model (Final KNPM): sales-weighted and raw results.

**Figure 2:**
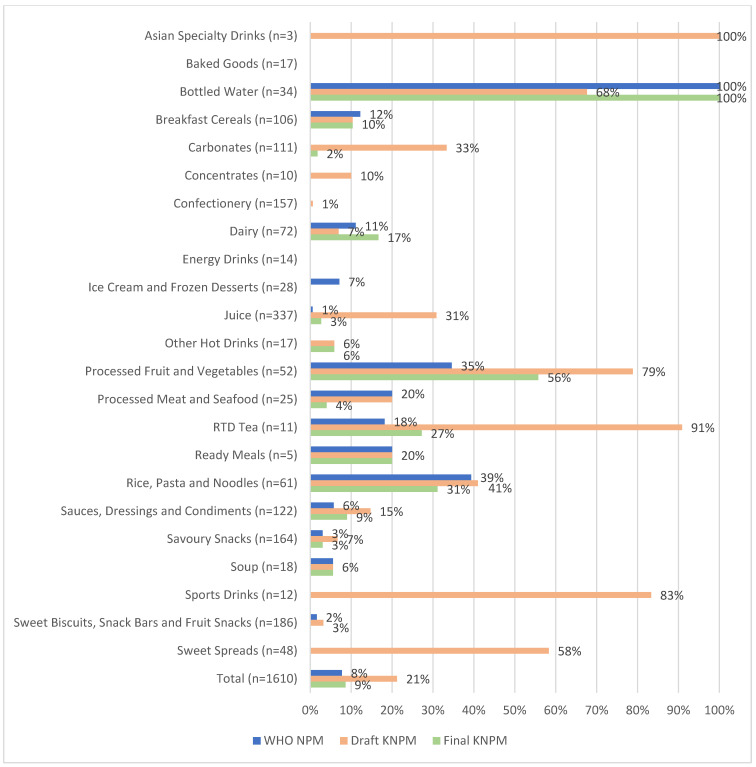
Proportion of products eligible under the WHO Nutrient Profile Model (WHO NPM), draft Kenyan Nutrient Profile Model (Draft KNPM), and final Kenyan Nutrient Profile Model (Final KNPM) (unweighted/raw results).

Figure 1 shows the unweighted and weighted results overall for the proportion of products eligible under each NPM. The proportion of eligible products increased when results were weighted by sales for both the WHO NPM (8% to 15%) and final KNPM (9% to 17%), indicating that healthier products represented a larger proportion of sales. The opposite trend was seen with the draft KNPM, where the proportion of eligible products was lower when weighted by sales (21% to 15%). Figure 2 shows the proportion of products eligible under each NPM both overall and by category. The draft KNPM had the most eligible products in most beverage categories, such as *RTD Tea*, *Carbonates*, *Concentrates*, *Asian Specialty Drinks* and *Sports Drinks*. The final KNPM allowed for a higher proportion of eligible *Dairy* products, with the WHO NPM having a higher proportion of eligible products in the *Breakfast Cereals* category compared to the draft and final KNPMs.

**Figure 3:**
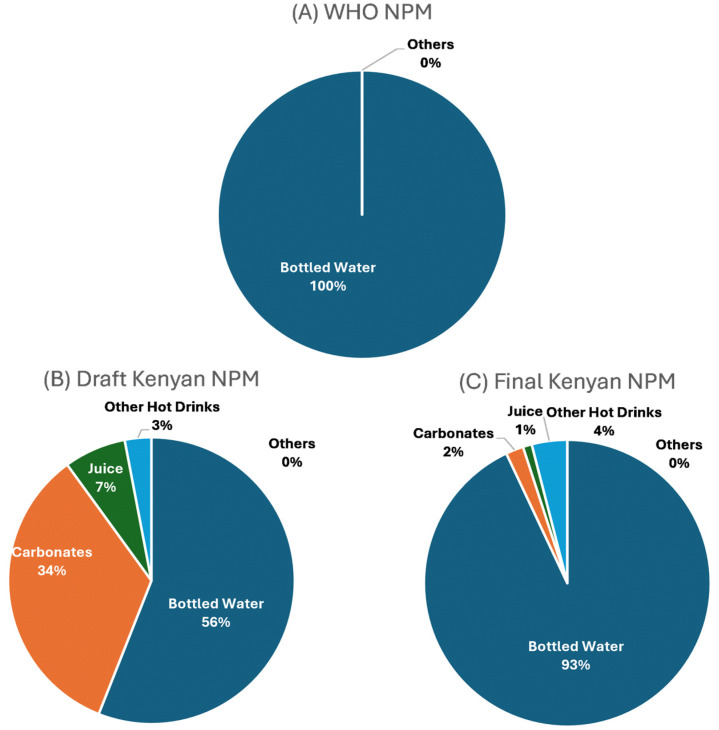
Proportion of sales-weighted beverage products eligible under the WHO Nutrient Profile Model (WHO NPM), draft Kenyan Nutrient Profile Model (Draft Kenyan NPM), and final Kenyan Nutrient Profile Model (Final Kenyan NPM).

Figure 3 shows the differences in the sales-weighted proportions of beverage products eligible under each NPM. Results were weighted by total beverage sales (excluding all food sales), and results varied across the three different models. *Bottled Water* represented the highest proportion of healthy beverage sales under all models. Under the draft KNPM, the *Carbonates* category had the highest sales-weighted proportion of products eligible (34%) after *Bottled Water*, due to this model having the most lenient criteria. *Bottled Water* represented a higher proportion of healthy beverage sales under the WHO NPM (100%) compared to the final KNPM (93%) and draft KNPM (56%). Interestingly, under both the draft and final KNPMs *Carbonates* represented 34% and 2%, respectively, of healthy beverage sales, compared to 0% under the WHO NPM. This is due to the WHO NPM not allowing carbonated beverages containing any sugar or non-nutritive sweeteners to be eligible for marketing to children, with the draft KNPM not including criteria for non-nutritive sweeteners and the final KNPM allowing products with up to 5.3 g/100 mL of total sugar to be eligible.

Figure S1 shows the differences in the sales-weighted proportions of food products eligible under each NPM. Results were weighted by total food sales (excluding all beverage sales), and results varied across the three different models. For all three models, the *Dairy* category had the highest sales-weighted proportion of food products eligible. The highest proportion was seen in the final KNPM (75%), followed by the WHO NPM (61%) and the draft KNPM (47%). The draft KNPM had a higher proportion of *Rice, Pasta and Noodles* (39%) compared to the final KNPM (20%) and the WHO NPM (30%). Few other substantial differences were seen for food categories between the three models.

### 3.3. Agreement Between the Three Nutrient Profile Models

Overall, there was 82% agreement between all three NPMs (*k* = 0.44; Table 2), indicating moderate agreement. Highest agreement was seen between the WHO NPM and the final KNPM (95%; *k* = 0.66), followed by the draft vs. final KNPM (85%; *k* = 0.43) and then the WHO NPM vs. the draft KNPM (83%; *k* = 0.35). Using all three models, there was 100% agreement for *Baked Goods*, *Energy Drinks*, *Ready Meals* and *Soup* and the lowest agreement for *Asian Specialty Drinks* (0%), *Sports Drinks* (17%) and *RTD Tea* (27%). There was ≥90% agreement for 19/23 categories between the WHO NPM and the final KNPM. There was a slightly larger proportion of Kenyan products included in the final comparison analysis (56%) compared with imported (44%; Table S1), with a higher proportion of imported products in categories such as *Sweet Biscuits*, *Sweet Spreads*, *Sauces* and *Soups*. In contrast, a much higher proportion of products in categories such as *Juice*, *Ice Cream*, *Bottled Water* and *RTD Tea* were locally produced.

## 4. Discussion

This research showcased some important findings that will be critical in informing policies related to the healthiness of packaged food and beverage products in Kenya, and indeed throughout the African region. The rise in diet-related non-communicable diseases in Africa is strongly linked to changes in dietary patterns and increasing consumption of processed packaged foods high in salt, saturated and trans fats and added sugars [3]. Kenya’s shift toward packaged and ultra-processed foods is driven by structural factors such as urbanization, lifestyle changes, and economic growth that have transformed food environments, with COVID-19 further accelerating supermarket and online shopping [13,14,15]. Nearly 80% of Kenyans cannot afford a healthy diet, while ultra-processed foods remain cheaper, more accessible, and heavily marketed [16]. Food-safety concerns, including chemical contamination in fresh produce, also push consumers toward packaged foods seen as safer [13,14,17,18]. Moreover, these are perceived as modern, urban, classy and appealing to young people [19], which can also contribute to the increase in the consumption of such foods.

Many countries within the African region now face rapid supermarketization, a growing incidence of NCDs, and often have weak labelling regulations, making evaluations of the healthiness of their food supplies and the implementation of a national NPM difficult. With Kenya having one of the most prospering supermarket sectors in sub-Saharan Africa [5], it is a hugely important step that the Kenyan government has launched the region’s first national NPM. The results of this study examining various regionally applicable models will therefore be useful in informing broader work in the region, and can help inform policies in other regions of the world that may face similar challenges to Africa.

There is only a small number of studies examining the nutrient content of Kenyan foods prior to this study being undertaken. One study has examined the sodium content of Kenyan packaged food and beverage products and found that only 39% of all products displayed sodium on the label (almost exactly matching our finding of 40% in the current study), and that imported products were more likely than Kenyan/locally produced products to display sodium on pack [20]. More recently, a report by the Access to Nutrition Initiative released in July 2025 found that 10% of products (15% sales-weighted) sold by the 30 top-selling Kenyan manufacturers met the nutritional criteria of a pre-final version of the KNPM and that 14% (23% sales-weighted) of products met the criteria for marketing to children established by the WHO NPM [21]. These findings differ slightly from the present study, with the present study using products from all available manufacturers and using the final version of the KNPM, finding 9% (18% sales-weighted) of products meeting the final KNPM criteria and 8% (15% sales-weighted) meeting the WHO NPM criteria.

Our study also demonstrated a very low proportion of products adhering to minimum Codex labelling requirements (21%). The fact that a higher proportion of imported products adhered to minimum Codex labelling requirements, as well as labelling additional nutrients of public health concern, such as trans fat, poses the question as to whether Kenyan labelling requirements are on par with those of other countries around the world. Since locally produced products are often more affordable and more widely purchased by lower-income households, this labelling gap raises a clear equity concern. Consumers with the least purchasing power are also those least likely to receive complete nutrition information, limiting their ability to make informed dietary choices and potentially widening health inequities. Similarly, ensuring harmonization between Kenya’s nutrient declaration regulations and Codex guidelines would better facilitate not only the implementation and monitoring of the KNPM but would likely also facilitate trade and compliance between countries in the region (and globally).

Large differences were observed when evaluating beverages under the draft KNPM and the final KNPM. These differences were primarily positive as the final KNPM was more restrictive than the draft KNPM. For example, 31% of *Juice* products were eligible under the draft KNPM, dropping to only 3% under the final KNPM. Similarly, the proportion of *Carbonates* eligible decreased from 33% to 2%. This is a particularly important discussion point, seeing as carbonates represented over KES 55 billion (USD $425.5 million) of Kenyan consumer spending in 2021 (compared to, for example, confectionery with KES 10.5 billion [USD $81 million] and juice with just over 13 billion [USD $100.6 million]). It may be that additional nutrient criteria for beverage products, particularly soft drinks, are warranted. For example, the WHO NPM does not allow any soft drink products that contain added sugar or sweeteners to be eligible. In contrast, the final KNPM allows products with up to 5.3 g of sugar per 100 mL to be eligible. The draft KNPM resulted in a higher proportion of sales-weighted beverage products from the juice category being eligible compared to the WHO NPM and final KNPM. This is due to the draft KNPM allowing juices with up to 10 g of sugar per 100 mL to be eligible, versus 6 g per 100 mL in the WHO NPM and 5.3 g per 100 mL in the final KNPM. Long-term overconsumption of fructose (the sugar found in fruit juice) has been linked to cardiovascular and metabolic diseases [22] and, as such, other NPMs in use around the world place restrictions on marketing of fruit juice products to children.

Many previous studies have examined agreement between various regionally or nationally appropriate NPMs, with this being the first in Kenya. The present study found moderate agreement (82%: *k* = 0.44) between all three NPMs, but substantial agreement between the WHO NPM and the final KNPM (95%; *k* = 0.66). It is difficult to directly place the results of this study within the existing literature, as results are highly dependent on the type of NPMs being compared. The development of the final KNPM used the WHO NPM as its basis, and so considerable agreement is expected. This is certainly the case in studies in other markets examining agreement between NPMs in which one was developed off the back of another. For example, multiple studies examining agreement between the Health Star Rating and Nutri-Score have observed substantial agreement (*k* = 0.62 [23] and *k* = 0.78 [24]) between the two schemes, as both NPMs were developed on the basis of the UK’s Ofcom NPM. Similarly, a study from Saudi Arabia observed substantial agreement (*k* = 0.74) when comparing the Saudi Arabian NPM and Nutri-Score [25], with development of both also heavily based on the UK’s Ofcom NPM. The present study is the first to examine adherence of products to either the WHO NPM or the KNPM and will serve as a useful baseline for future research and monitoring.

This study has limitations. Data from Innova Market Insights are subject to errors, and although efforts were made to remove erroneous data from analyses, there is always the chance that some data were incorrect. Additionally, data from Innova Market Insights provides information for food and beverages that are either new to supermarket shelves or that are products that have been reformulated, and so it is possible that some products that have remained on the market in Kenya for beyond the ten years used in this analysis. A similar limitation with the use of Innova data is that there is a chance that the database may overrepresent newly launched or reformulated products, and as such, it is unknown whether the launch of newer, potentially more innovative products (and hence underrepresentation of long-standing, low-cost brands) may have affected overall results. Euromonitor sales data are not provided for every individual product and instead can only be used to estimate overall category-level sales. There is also no information available to help understand what coverage of products the Innova dataset represents in Kenya. Due to such a low proportion of products labelling key nutrients required for all the NPMs examined, the number of products able to be included in the analysis is much smaller than what is available on supermarket shelves. This level of data missingness is not unusual in countries without strong labelling legislation, with similar studies undertaken in Kenya reporting high levels of data missingness when applying nutrient profile models (varying depending on which NPM is applied) [20,21]. Additionally, foods and beverages purchased and consumed outside the home, such as from fast food outlets or restaurants, were not included in this study but represent an important component for consideration in future research and food policies in Kenya, particularly given that research has shown that intake of these foods is increasing rapidly across Africa [6]. The limited research available suggests that between 2002 and 2018, fast food chain sales in Kenya increased by 70% [6], which is in line with data from Euromonitor International, which has shown some unhealthy food categories have had growth upwards of >70% between 2016 and 2021 [26].

## 5. Conclusions

This study provides the first comprehensive assessment of nutrition labelling practices and nutrient profiling of packaged food and beverages in Kenya. Labelling compliance remains low, with only 21% of products meeting minimum Codex requirements and 4% including key nutrients of public health concern. Imported products were more likely than domestic products to provide full nutrition information, highlighting a significant equity gap in consumer access to information. From our study, several priorities emerged. First, mandatory nutrition labelling for domestic and imported packaged food—both back-of-pack and interpretive front-of-pack—is essential to ensure consumers have access to accurate information. Government policies should ensure local regulations insist that a minimum set of nutrients appear on all packaged food and beverage product labels, and/or only allow front-of-pack labelling on products that also provide sufficient back-of-pack information.

Second, monitoring and enforcement mechanisms must be established to ensure that the KNPM is implemented consistently, reviewed regularly, and adapted as the food supply and global evidence evolve. Third, the absence of data on fast food and restaurant products remains a critical evidence gap; given rising sales, these should be included in future surveillance.

## Figures and Tables

**Table 1:** Proportion of products in Kenya displaying minimum Codex nutrients as well as nutrients of public health concern.

	Proportion of Products with Nutrients Labelled On-Pack								% Labelled	
	Total Fat	Carbohydrate	Energy	Protein	Saturated Fat	Sugars	Sodium	Trans Fat	Codex	All
Baby Food (*n* = 76)	74	76	83	78	29	39	63	0	74	0
Baked Goods (*n* = 546)	14	16	13	16	10	9	10	4	10	0
Bottled Water (*n* = 40)	18	15	3	15	18	18	63	0	0	0
Breakfast Cereals (*n* = 172)	85	88	76	90	64	69	70	26	72	14
Carbonates (*n* = 119)	92	87	92	85	82	69	93	2	82	1
Concentrates (*n* = 44)	25	32	32	23	25	11	23	0	20	0
Confectionery (*n* = 426)	65	65	54	64	53	54	56	12	53	3
Dairy (*n* = 589)	66	76	68	76	15	20	16	3	54	1
Edible Oils (*n* = 57)	65	56	63	54	56	51	26	28	49	2
Energy Drinks (*n* = 20)	90	85	100	85	90	85	70	0	80	0
Ice Cream (*n* = 140)	59	59	51	59	29	45	26	9	51	1
Juice (*n* = 374)	78	87	84	84	51	44	47	10	69	6
Other Hot Drinks (*n* = 276)	25	33	25	29	16	17	20	3	17	0
Processed Fruit/Vegetables (*n* = 118)	68	69	54	68	53	52	58	19	53	9
Processed Meat and Seafood (*n* = 111)	38	36	35	38	26	27	26	5	33	0
RTD Tea (*n* = 21)	67	48	71	67	62	57	67	0	67	0
Ready Meals (*n* = 109)	11	20	18	20	8	6	13	5	7	0
Rice, Pasta and Noodles (*n* = 226)	62	61	66	64	34	32	33	14	56	10
Sauces/Dips/Condiments (*n* = 587)	36	36	25	36	28	31	34	12	23	4
Savoury Snacks (*n* = 628)	64	64	53	64	40	40	52	17	52	6
Soup (*n* = 21)	100	100	95	100	86	95	95	19	95	14
Sports Drinks (*n* = 13)	69	92	46	62	69	77	92	38	15	0
Sweet Biscuits (*n* = 717)	84	81	67	84	48	58	51	28	66	8
Sweet Spreads (*n* = 133)	68	71	52	68	47	42	48	17	45	4
Total (*n* = 5587)	57%	59%	52%	59%	36%	37%	39%	12%	46%	4%

**Table 2:** Agreement between the WHO NPM, draft KNPM and final KNPM.

					Agreement							
Category		WHO	DKNPM	FKNPM	All 3 NPMs		WHO vs. Draft		WHO vs. Final		Draft vs. Final	
	N	% Eligible	% Eligible	% Eligible	% Agreement	Kappa	% Agreement	Kappa	% Agreement	Kappa	% Agreement	Kappa
Asian Drinks	3	0%	100%	0%	0%	−0.50	0%	-	100%	1.00	0%	0.00
Baked Goods	17	0%	0%	0%	100%	1.00	100%	1.00	100%	1.00	100%	1.00
Bottled Water	34	100%	68%	100%	68%	−0.12	68%	0.00	100%	1.00	68%	0.00
Breakfast Cereals	106	12%	10%	10%	98%	0.94	98%	0.91	98%	0.91	100%	1.00
Carbonates	111	26%	33%	2%	67%	−0.07	67%	0.00	98%	0.00	68%	0.07
Concentrates	10	0%	10%	0%	90%	−0.03	90%	0.00	100%	1.00	90%	0.00
Confectionery	157	0%	1%	0%	99%	0.00	99%	0.00	100%	1.00	99%	0.00
Dairy	72	11%	7%	17%	79%	0.32	90%	0.41	81%	0.19	88%	0.41
Energy Drinks	14	0%	0%	0%	100%	1.00	100%	1.00	100%	1.00	100%	1.00
Ice Cream	28	7%	0%	0%	93%	−0.02	93%	0.00	93%	0.00	100%	1.00
Juice	337	59%	31%	3%	69%	−0.02	69%	0.01	97%	0.17	72%	0.12
Other Hot Drinks	17	0%	6%	6%	94%	0.48	94%	0.00	94%	0.00	100%	1.00
Processed Fruit/Vegetables	52	35%	79%	56%	52%	0.35	56%	0.25	71%	0.44	77%	0.51
Processed Meat/Seafood	25	20%	20%	4%	84%	0.57	100%	1.00	84%	0.29	84%	0.29
RTD Tea	11	18%	91%	27%	27%	0.02	27%	0.04	91%	0.74	36%	0.07
Ready Meals	5	20%	20%	20%	100%	1.00	100%	1.00	100%	1.00	100%	1.00
Rice/Pasta/Noodles	61	39%	41%	31%	90%	0.86	98%	0.97	92%	0.82	90%	0.79
Sauces	122	6%	15%	9%	84%	0.41	86%	0.26	89%	0.16	94%	0.73
Savoury Snacks	164	3%	7%	3%	93%	0.40	95%	0.48	94%	−0.03	96%	0.61
Soup	18	6%	6%	6%	100%	1.00	100%	1.00	100%	1.00	100%	1.00
Sports Drinks	12	0%	83%	0%	17%	−0.38	17%	0.00	100%	1.00	17%	0.00
Sweet Biscuits	186	2%	3%	0%	97%	0.32	98%	0.66	98%	0.00	97%	0.00
Sweet Spreads	48	0%	58%	0%	42%	−0.24	42%	0.00	100%	1.00	42%	0.00
Total	1610	8%	21%	9%	82%	0.44	83%	0.35	95%	0.66	85%	0.43

## Data Availability

Data described in the manuscript, code book, and analytic code will not be made available because of the proprietary nature of the Innova data.

## Supplementary Materials

The following supporting information can be downloaded at: https://www.mdpi.com/article/10.3390/nu18040566/s1, Figure S1: Proportion of food sales deriving from eligible products under each nutrient profile model; Table S1: Number and proportion of products containing sufficient and insufficient nutrient data to apply the three nutrient profile models, by category; Table S2: Proportion of imported products available in Kenya displaying minimum CODEX nutrients as well as nutrients of public health concern; Table S3: Proportion of domestic products available in Kenya displaying minimum CODEX nutrients as well as nutrients of public health concern.

## References

[1] Forouzanfar M.H., Alexander L., Anderson H.R., Bachman V.F., Biryukov S., Brauer M., Burnett R., Casey D., Coates M.M., Cohen A. (2015). Global, regional, and national comparative risk assessment of 79 behavioural, environmental and occupational, and metabolic risks or clusters of risks in 188 countries, 1990–2013: A systematic analysis for the Global Burden of Disease Study 2013. Lancet.

[2] Malhotra A., Noakes T., Phinney S. (2015). It is time to bust the myth of physical inactivity and obesity: You cannot outrun a bad diet. Br. J. Sports Med..

[3] Prentice A.M. (2006). The emerging epidemic of obesity in developing countries. Int. J. Epidemiol..

[4] Rischke R., Kimenju S.C., Klasen S., Qaim M. (2015). Supermarkets and food consumption patterns: The case of small towns in Kenya. Food Policy.

[5] Demmler K.M., Ecker O., Qaim M. (2018). Supermarket Shopping and Nutritional Outcomes: A Panel Data Analysis for Urban Kenya. World Dev..

[6] Reardon T., Tschirley D., Liverpool-Tasie L.S.O., Awokuse T., Fanzo J., Minten B., Vos R., Dolislager M., Sauer C., Dhar R. (2021). The processed food revolution in African food systems and the double burden of malnutrition. Glob. Food Secur..

[7] World Health Organization (2017). Tackling NCDs: ‘Best Buys’ and Other Recommended Interventions for the Prevention and Control of Noncommunicable Diseases.

[8] Sacks G., Rayner M., Stockley L., Scarborough P., Snowdon W., Swinburn B. (2011). Applications of nutrient profiling: Potential role in diet-related chronic disease prevention and the feasibility of a core nutrient-profiling system. Eur. J. Clin. Nutr..

[9] World Health Organzation (2019). Nutrient Profile Model for the African Region.

[10] Ministry of Health (2025). Kenya Nutrient Profile Model.

[11] Asiki G., Wanjohi M.N., Barnes A., Bash K., Muthuri S., Amugsi D., Doughman D., Kimani E., Vandevijvere S., Holdsworth M. (2020). Benchmarking food environment policies for the prevention of diet-related non-communicable diseases in Kenya: National expert panel’s assessment and priority recommendations. PLoS ONE.

[12] Innova Market Insights. https://www.innovamarketinsights.com/.

[13] Jumia (2020). Jumia Food. Africa Food Index.

[14] Unicef Kenya (2025). An Analysis of Food Systems for Children in Kenya.

[15] Unicef Kenya (2025). Enhancing Kenya’s Food System to Prevent and Address Child & Adolescent Malnutrition.

[16] De Jong M.V., Selten M.P.H., Gitata-Kiriga W., Peters B., Dengerink J.D. (2024). An Overview of the Kenyan Food System: Outcomes, Drivers and Activities.

[17] Pradeilles R., Irache A., Wanjohi M.N., Holdsworth M., Laar A., Zotor F., Tandoh A., Klomegah S., Graham F., Muthuri S.K. (2021). Urban physical food environments drive dietary behaviours in Ghana and Kenya: A photovoice study. Health Place.

[18] Consumer Grassroots Association (2021). Food Safety in Kenya, A Consumer Perspective.

[19] Wanjohi M.N., Kimani-Murage E.W., Holdsworth M., Pradeilles R., Wilunda C., Asiki G., Klipstein-Grobusch K. (2025). Drivers and solutions to unhealthy food consumption by adolescents in urban slums, Kenya: A qualitative participatory study. Public Health Nutr..

[20] Ndanuko R., Maganja D., Kibet A., Coyle D.H., Kimiywe J., Raubenheimer D., Marklund M., Wu J.H.Y. (2021). Sodium Content and Labelling Completeness of Packaged Foods and Beverages in Kenya. Nutrients.

[21] Access to Nutrition Initiative Report on the Comparative Nutritional Profile of 983 Food and Beverage Products Marketed by 30 Large Companies Operating in Kenya. https://accesstonutrition.org/app/uploads/2025/06/20250630_EAMA_Kenya_Full_TGI_PP_2025_FINAL.pdf.

[22] Giussani M., Lieti G., Orlando A., Parati G., Genovesi S. (2022). Fructose Intake, Hypertension and Cardiometabolic Risk Factors in Children and Adolescents: From Pathophysiology to Clinical Aspects. A Narrative Review. Front. Med..

[23] Hafner E., Pravst I. (2023). Comparison of Nutri-Score and Health Star Rating Nutrient Profiling Models Using Large Branded Foods Composition Database and Sales Data. Int. J. Environ. Res. Public Health.

[24] Dickie S., Woods J., Machado P., Lawrence M. (2022). Nutrition Classification Schemes for Informing Nutrition Policy in Australia: Nutrient-Based, Food-Based, or Dietary-Based?. Curr. Dev. Nutr..

[25] Dunford E.K., Alsukait R.F., Alkhalaf M.M., Hamza M.M., Shahin M.A., Cetinkaya V., Alghaith T. (2025). The Healthiness of Packaged Food and Beverage Products in the Kingdom of Saudi Arabia. Nutrients.

[26] Euromonitor International Euromonitor Passport. https://www.euromonitor.com/solutions/passport.

