# Supplementary material for: Nutrition Labelling Practices and the Healthiness of Packaged Food and Beverage Products Available in Kenya
Source: Nutrients. 2026 Feb 9;18(4):566. doi: 10.3390/nu18040566 (PMC12942844; doi:10.3390/nu18040566)
Supplement: Supplementary file 1 [file nutrients-18-00566-s001.zip › nutrients-4016906-supplementary.pdf]

Supplementary Table S1: Number and proportion of products containing sufficient nutrient data to apply all three nutrient profile models, by category and import status

	N (%) included in final analysis	% final products (domestic)	% final products (imported)
**Asian Specialty Drinks (n=7)**	3 (43%)	67%	33%
**Baked Goods (n=248)**	17 (7%)	71%	29%
**Bottled Water (n=40)**	34 (85%)	100%	0%
**Breakfast Cereals (n=172)**	106 (62%)	54%	46%
**Carbonates (n=119)**	111 (93%)	68%	32%
**Concentrates (n=44)**	10 (23%)	100%	0%
**Confectionery (n=426)**	157 (37%)	42%	58%
**Dairy (n=589)**	72 (12%)	53%	47%
**Energy Drinks (n=20)**	14 (70%)	79%	21%
**Ice Cream (n=140)**	28 (20%)	82%	18%
**Juice (n=374)**	337 (90%)	80%	20%
**Other Hot Drinks (n=70)**	17 (24%)	29%	71%
**Processed Fruit/ Vegetables (n=118)**	52 (44%)	37%	63%
**Processed Meat and Seafood (n=111)**	25 (23%)	60%	40%
**RTD Tea (n=21)**	11 (52%)	100%	0%
**Ready Meals (n=109)**	5 (5%)	60%	40%
**Rice, Pasta and Noodles (n=226)**	61 (27%)	43%	57%
**Sauces/Dips/Condiments (n=271)**	122 (45%)	35%	65%
**Savoury Snacks (n=627)**	164 (26%)	68%	32%
**Soup (n=21)**	18 (86%)	22%	78%
**Sports Drinks (n=13)**	12 (92%)	50%	50%
**Sweet Biscuits (n=717)**	186 (26%)	27%	73%
**Sweet Spreads (n=133)**	48 (36%)	21%	79%
**Total (n=4620)**	**1,610 (35%)**	**56%**	**44%**

Supplementary Table S2: Proportion of imported products available in Kenya displaying minimum CODEX nutrients as well as nutrients of public health concern

	Proportion of products with nutrients labelled on-pack								% labelled	
	Tot fat	Carbohydrate	Energy	Protein	Sat fat	Sugars	Sodium	Trans	CODEX	All
**Asian Specialty Drinks (n=1)**	100%	100%	0%	100%	100%	100%	100%	100%	0%	0%
**Baby Food (n=35)**	100%	100%	100%	100%	63%	69%	97%	0%	57%	0%
**Baked Goods (n=67)**	51%	51%	25%	48%	37%	34%	37%	25%	9%	0%
**Breakfast Cereals (n=56)**	100%	100%	71%	100%	91%	93%	95%	45%	61%	18%
**Carbonates (n=40)**	88%	88%	83%	88%	88%	73%	88%	3%	68%	0%
**Confectionery (n=175)**	80%	82%	65%	80%	75%	77%	75%	21%	51%	2%
**Dairy (n=76)**	82%	71%	76%	72%	49%	50%	58%	13%	38%	9%
**Edible Oils (n=8)**	75%	38%	75%	38%	63%	38%	38%	13%	38%	0%
**Energy Drinks (n=3)**	100%	67%	100%	100%	100%	100%	100%	0%	67%	0%
**Ice Cream and Frozen Desserts (n=20)**	100%	100%	80%	100%	55%	50%	50%	30%	25%	0%
**Juice (n=70)**	94%	89%	61%	91%	81%	74%	74%	31%	40%	9%
**Other Hot Drinks (n=40)**	53%	53%	30%	53%	38%	45%	48%	8%	20%	0%
**Processed Fruit and Vegetables (n=43)**	91%	91%	67%	91%	81%	79%	86%	40%	58%	23%
**Processed Meat and Seafood (n=19)**	68%	58%	74%	68%	53%	53%	63%	11%	42%	0%
**Ready Meals (n=3)**	100%	100%	67%	100%	67%	100%	100%	33%	33%	0%
**Rice, Pasta and Noodles (n=90)**	76%	74%	68%	78%	42%	47%	52%	13%	29%	7%
**Sauces, Dressings and Condiments (n=127)**	86%	81%	54%	83%	72%	76%	86%	31%	43%	9%
**Savoury Snacks (n=104)**	99%	97%	67%	97%	80%	85%	95%	38%	40%	13%
**Soup (n=17)**	100%	100%	94%	100%	82%	94%	94%	24%	82%	18%
**Sports Drinks (n=6)**	100%	100%	17%	83%	100%	100%	100%	83%	0%	0%
**Sweet Biscuits, Snack Bars and Fruit Snacks (n=342)**	94%	89%	64%	94%	73%	86%	81%	45%	34%	9%
**Sweet Spreads (n=64)**	72%	77%	41%	70%	69%	70%	69%	23%	31%	5%
**Total (n=1406)**	86%	83%	63%	84%	69%	73%	75%	29%	40%	7%

Notes: data for Bottled Water, Concentrates, Eggs, RTD Coffee and RTD Tea not shown as there were zero imported products in these categories. “Imported” is defined as any product that is produced in a country ither than Kenya.

Supplementary Table S3: Proportion of domestic products available in Kenya displaying minimum CODEX nutrients as well as nutrients of public health concern

	Proportion of products with nutrients labelled on-pack								% labelled	
	Tot fat	Carbohydrate	Energy	Protein	Sat fat	Sugars	Sodium	Trans	CODEX	All
**Asian Specialty Drinks (n=6)**	83%	83%	67%	50%	67%	33%	33%	0%	33%	0%
**Baby Food (n=41)**	61%	56%	68%	68%	0%	15%	34%	0%	0%	0%
**Baked Goods (n=479)**	10%	12%	11%	12%	6%	5%	7%	1%	3%	0%
**Bottled Water (n=40)**	95%	15%	3%	93%	95%	95%	63%	0%	0%	0%
**Breakfast Cereals (n=116)**	78%	83%	78%	84%	51%	58%	58%	17%	46%	12%
**Carbonates (n=79)**	99%	87%	97%	84%	81%	71%	96%	1%	46%	1%
**Concentrates (n=44)**	32%	32%	32%	23%	32%	11%	23%	0%	9%	0%
**Confectionery (n=251)**	54%	53%	47%	53%	38%	39%	43%	6%	26%	3%
**Dairy (n=513)**	69%	77%	67%	77%	10%	16%	10%	2%	6%	0%
**Edible Oils (n=49)**	63%	59%	61%	57%	55%	53%	24%	31%	16%	12%
**Eggs (n=16)**	6%	6%	6%	6%	0%	0%	0%	0%	0%	0%
**Energy Drinks (n=17)**	100%	88%	100%	82%	100%	88%	65%	0%	47%	0%
**Ice Cream and Frozen Desserts (n=120)**	52%	52%	47%	52%	25%	44%	22%	5%	16%	1%
**Juice (n=304)**	91%	87%	90%	84%	57%	39%	41%	6%	26%	6%
**Other Hot Drinks (n=236)**	22%	30%	25%	26%	14%	13%	15%	2%	4%	0%
**Processed Fruit and Vegetables (n=75)**	55%	56%	47%	55%	37%	36%	43%	8%	19%	1%
**Processed Meat and Seafood (n=92)**	32%	32%	27%	32%	21%	22%	18%	4%	16%	0%
**RTD Tea (n=21)**	67%	48%	71%	67%	62%	57%	67%	0%	24%	0%
**Ready Meals (n=106)**	10%	19%	17%	19%	7%	4%	11%	4%	1%	0%
**Rice, Pasta and Noodles (n=136)**	59%	57%	65%	60%	29%	22%	21%	15%	18%	13%
**Sauces, Dressings and Condiments (n=460)**	23%	24%	17%	24%	16%	19%	21%	6%	9%	2%
**Savoury Snacks (n=524)**	57%	58%	50%	58%	32%	31%	46%	13%	20%	5%
**Soup (n=4)**	100%	100%	100%	100%	100%	100%	100%	0%	100%	0%
**Sports Drinks (n=7)**	86%	86%	71%	43%	86%	57%	86%	0%	29%	0%
**Sweet Biscuits, Snack Bars and Fruit Snacks (n=375)**	75%	75%	70%	76%	25%	33%	23%	12%	13%	5%

	Proportion of products with nutrients labelled on-pack								% labelled	
	Tot fat	Carbohydrate	Energy	Protein	Sat fat	Sugars	Sodium	Trans	CODEX	All
**Sweet Spreads (n=69)**	67%	70%	62%	70%	26%	19%	32%	10%	10%	3%
**Total (n=4181)**	51%	52%	48%	52%	26%	27%	27%	7%	14%	3%

"Domestic" is defined as any product that is produced in Kenya.

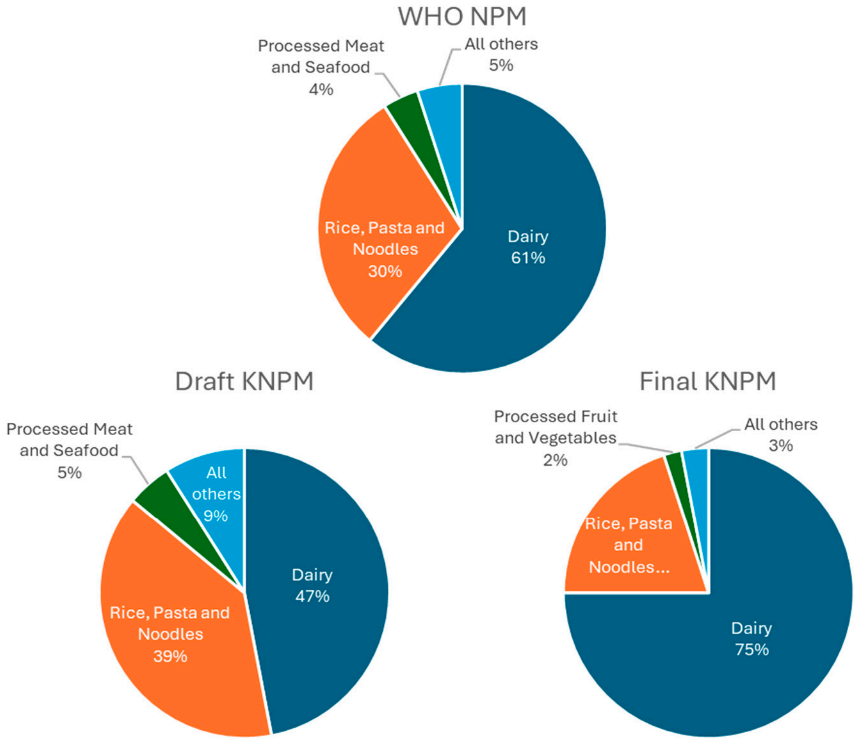

<u>**Supplementary Figure S1:**</u> Proportion of food sales deriving from eligible products under each nutrient profile model.
